# The impact of transient and persistent acute kidney injury in hospital mortality in COVID-19 patients

**DOI:** 10.1590/2175-8239-JBN-2021-0123

**Published:** 2021-12-03

**Authors:** João Bernardo, Joana Gonçalves, Joana Gameiro, João Oliveira, Filipe Marques, Inês Duarte, Carolina Branco, Claudia Costa, Carolina Carreiro, José Nuno Fonseca, Sandra Braz, José António Lopes

**Affiliations:** 1 Centro Hospitalar Lisboa Norte, Departamento de Nefrologia e Transplante Renal, Lisboa, Portugal.; 2 Universidade de Lisboa, Faculdade Medicina, Lisboa, Portugal.; 3 Centro Hospitalar Lisboa Norte, Departamento de Medicina Interna, Lisboa, Portugal.

**Keywords:** Acute Kidney Injury, COVID-19, SARS-CoV-2, Hospital Mortality, Injúria Renal Aguda, COVID-19, SARS-CoV-2, Mortalidade Hospitalar

## Abstract

**Introduction:**

Acute kidney injury (AKI) has been described in Coronavirus Disease 2019 (COVID-19) patients and is considered a marker of disease severity and a negative prognostic factor for survival. In this study, the authors aimed to study the impact of transient and persistent acute kidney injury (pAKI) on in-hospital mortality in COVID-19 patients.

**Methods:**

This was a retrospective observational study of patients hospitalized with COVID-19 in the Department of Medicine of the Centro Hospitalar Universitario Lisboa Norte, Lisbon, Portugal, between March 2020 and August 2020. A multivariate analysis was performed to predict AKI development and in-hospital mortality.

**Results:**

Of 544 patients with COVID-19, 330 developed AKI: 166 persistent AKI (pAKI), 164 with transient AKI. AKI patients were older, had more previous comorbidities, had higher need to be medicated with RAAS inhibitors, had higher baseline serum creatine (SCr) (1.60 mg/dL vs 0.87 mg/dL), higher NL ratio, and more severe acidemia on hospital admission, and more frequently required admission in intensive care unit, mechanical ventilation, and vasopressor use. Patients with persistent AKI had higher SCr level (1.71 mg/dL vs 1.25 mg/dL) on hospital admission. In-hospital mortality was 14.0% and it was higher in AKI patients (18.5% vs 7.0%). CKD and serum ferritin were independent predictors of AKI. AKI did not predict mortality, but pAKI was an independent predictor of mortality, as was age and lactate level.

**Conclusion:**

pAKI was independently associated with in-hospital mortality in COVID-19 patients but its impact on long-term follow-up remains to be determined.

## Resumo

**Introdução:**

A lesão renal aguda (LRA) foi descrita em pacientes com doença do Coronavírus 2019 (COVID-19) e é considerada um marcador de gravidade da doença e fator prognóstico negativo para sobrevivência. Neste estudo, os autores visaram estudar o impacto da lesão renal aguda transitória e persistente (LRAp) na mortalidade hospitalar em pacientes com COVID-19.

**Métodos:**

Estudo observacional retrospectivo de pacientes internados com COVID-19 no Departamento de Medicina do Centro Hospitalar Universitário Lisboa Norte, Lisboa, Portugal, entre Março-Agosto de 2020. Realizou-se análise multivariada para prever desenvolvimento de LRA e mortalidade hospitalar.

**Resultados:**

De 544 pacientes com COVID-19, 330 desenvolveram LRA: 166 LRA persistente (LRAp), 164, LRA transitória. Pacientes com LRA eram mais velhos, apresentaram mais comorbidades prévias, maior necessidade de serem medicados com inibidores do SRAA, apresentaram creatina sérica basal mais elevada (CrS) (1,60 mg/dL vs 0,87 mg/dL), maior razão NL, e acidemia mais grave na admissão hospitalar, e necessitaram mais frequentemente de internação na UTI, ventilação mecânica, e uso de vasopressores. Pacientes com LRA persistente apresentaram maior nível de CrS (1,71 mg/dL vs 1,25 mg/dL) na admissão hospitalar. A mortalidade hospitalar foi de 14,0% e foi maior em pacientes com LRA (18,5% vs 7,0%). A DRC e ferritina sérica foram preditores independentes de LRA. A LRA não previu mortalidade, mas a LRAp foi um preditor independente de mortalidade, assim como idade e nível de lactato.

**Conclusão:**

A LRAp foi associada independentemente à mortalidade hospitalar em pacientes com COVID-19, mas seu impacto no acompanhamento de longo prazo ainda precisa ser determinado.

## Introduction

In late December 2019, a surge of atypical severe pneumonia was detected in Wuhan, China. The initial cases were all associated with the local wholesale food market and caused by severe acute respiratory syndrome coronavirus 2 (SARS-CoV-2)^1^. This disease became known as Coronavirus Disease 2019 (COVID-19). The infection spread rapidly around the world and it was declared a pandemic by the World Health Organization on March 11, 2020^2^. Bu the end of January 2021, almost 100 million cases of COVID-19 had been reported worldwide, resulting in more than two million deaths^3^.

Most patients present with mild symptoms such as fever, dyspnea, cough, headache, and diarrhea or are asymptomatic. However, more severe cases of pneumonia can lead to acute respiratory distress syndrome (ARDS), septic shock, multiple organ failure, and death^4^^,^ 5.

Acute kidney injury (AKI) has been described in COVID-19 patients and is considered a marker of disease severity and a negative prognostic factor for survival^6^^,^ 7. However, which factors predict mortality in patients with COVID-19 with AKI is still unknown.

The authors studied the impact of transient and persistent acute kidney injury on in-hospital mortality in COVID-19 patients.

## Patients and Methods

This study was a retrospective analysis of hospitalized patients admitted to the Dedicated Unit for COVID-19 patients (UICIVE) at the Department of Medicine of the Centro Hospitalar Universitario Lisboa Norte (CHULN), in Lisbon, Portugal, between March and August, 2020. The Ethical Committee approved this study in agreement with institutional guidelines and informed consent was waived, given its retrospective and non-interventional nature of the study.

Eligible patients were adults (≥18 years of age) who tested positive for COVID-19 by polymerase chain reaction testing of a nasopharyngeal sample and were admitted at the UICIVE from March 1st to May 31st of 2020. For patients who had multiple qualifying hospital admissions, we included only the first hospitalization. Exclusion criteria were (a) chronic kidney disease (CKD) patients on renal replacement therapy, (b) patients who underwent renal replacement therapy one week prior to admission, (c) patients who had less than 2 determinations of SCr and (d) patients who were discharged or died less than two days after admission.

Data were collected from individual electronic clinical records. The following variables were analyzed: demographic characteristics (age, gender); comorbidities [diabetes mellitus, hypertension, chronic obstructive pulmonary disease (COPD), cardiovascular disease (CVD), cirrhosis, CKD and/or active malignancy]; current treatment with RAAS inhibitors; disease severity according to the Brescia-COVID Respiratory Severity Scale (BCRSS) on admission^8^; laboratory values on admission [serum hemoglobin, hematocrit, neutrophil, lymphocyte and platelet count, serum albumin, serum ferritin, serum creatinine, C-reactive protein (CRP), arterial blood gas and pH analysis, and lactic acid dehydrogenase (LDH)]; exposure to nephrotoxins during the first week of admission [non-steroidal anti-inflammatory drugs (NSAIDS), radiocontrast, vancomycin, aminoglycosides]; need for intensive care unit (ICU) admission; need for mechanical ventilation; vasopressor use; and treatment for COVID-19 (hydroxychloroquine, lopinavir/ritonavir, corticosteroids, tocilizumab, remdesivir). Diagnosis of COVID-19 was based on the WHO interim guidelines^9^.

AKI that developed during hospital stay was defined according to the Kidney Disease Improving Global Outcomes (KDIGO) classification, using the serum creatinine (SCr) criteria, as follows: Stage 1: increase in SCr by 0.3 mg/dL within 48 hours or a 1.5-1.9-fold increase in SCr from baseline within 7 days; Stage 2: 2.9-fold increase in SCr within 7 days; Stage 3: 3-fold or greater increase in SCr within 7 days or initiation of renal replacement therapy (RRT)^10^. Patients were stratified according to the highest AKI stage reached during their hospital stay. Persistent AKI (pAKI) was defined as continued AKI beyond 48 h according to the KDIGO criteria as per the consensus report of the ADQI 16 Workgroup^11^. Transient AKI (tAKI) was defined as AKI of less than 48 h duration^11^.

Pre-admission SCr (SCr within the previous three months) was considered as baseline value. The estimated glomerular filtration rate (eGFR) for patients with previous baseline SCr was calculated using the Chronic Kidney Disease Epidemiology Collaboration (CKD-EPI) creatinine equation^12^.When unavailable, baseline SCr was estimated from the MDRD equation, accepting the lower limit of a normal baseline GFR of 75 mL/min/1.73 m^2^, as previously proposed^10^. Presence of CKD was estimated according to the baseline SCR as an eGFR of lower than 60 mL/min/1.73 m^(2 13,25)^.

Diabetes mellitus was diagnosed according to the American Diabetes Association criteria^14^. Hypertension was diagnosed according to the 2018 European Society of Cardiology (ESC) and European Society of Hypertension Guidelines^15^.COPD comprised emphysema and chronic bronchitis. CVD was considered whenever a history of cerebrovascular disease, chronic heart failure of any cause, cardiac ischemic disease, and/or peripheral arterial disease was documented. Acidemia was defined as blood gas pH <7.35. N/L ratio at admission was calculated as: neutrophil count / lymphocyte count.

Analyzed outcomes were the development of AKI during the first week of admission and in-hospital mortality.

### Statistical analysis

Categorical variables were described as the total number and percentage for each category, whereas continuous variables were described as the mean ± standard deviation. Continuous variables were compared with the Student's t-test and categorical variables were compared with the Chi-square test. All variables underwent univariate analysis to determine statistically significant factors that may have contributed to AKI development and in-hospital mortality. Subsequently, significant variables were included in the multivariate analysis using the logistic regression method. Data were reported as odds ratios (ORs) with 95% confidence intervals (CIs). Statistical significance was defined as a P-value <0.05. Statistical analysis was performed with the SPSS for windows statistical software package (version 21.0).

## Results

### Participants

From March 1st to August 30st, 544 patients were admitted to UICIVE with a diagnosis of COVID-19. Patients' demographic and clinical data are described in Table 1.

**Table 1:** Patients’ baseline characteristics according to AKI development

Characteristic	Total (n=544)	Non-AKI (n=214)	AKI (n=330)	p- value
Age (year)	68.9±17.9	64.23±18.38	71.73±17.03	<0.001
Gender (male) – n (%)	298 (54.8)	107 (50.0)	191 (57.9)	0.346
Co-morbidities – n (%)				
Hypertension	345 (63.4)	107 (50.0)	238 (72.1)	<0.001
Diabetes	146 (26.8)	43 (20.0)	103 (31.2)	0.016
CVD	175 (32.2)	46 (21.5)	129 (39.1)	<0.001
CKD	103 (18.9)	17 (7.9)	86 (26.1)	<0.001
COPD	69 (12.7)	26 (12.1)	43 (13.0)	1.000
Cirrhosis	25 (4.6)	11 (5.1)	14 (4.2)	0.597
Neoplasia	87 (16.0)	34 (15.9)	53 (16.1)	0.769
RAAS inhibitors – n (%)	226 (41.5)	69 (32.2)	157 (47.6)	0.002
Baseline SCr (mg/dL)	0.98±0.44	0.89±0.42	1.03±0.44	<0.001
Baseline eGFR (mL/min/1.73m^2^)	75.68±24.89	83.50±23.38	70.96±24.62	<0.001
Brescia Score ≥ 2	78 (14.3)	22 (10.3)	56 (32.9)	0.039
Laboratory				
Admission SCr (mg/dL)	1.32±1.47	0.87±0.55	1.60±1.76	<0.001
Hemoglobin (g/dL)	12.68±2.29	12.82±2.22	12.59±2.34	0.242
Anemia – n (%)	224 (41.2)	80 (37.4)	144 (43.6)	0.455
NL ratio	6.36±6.28	5.15±4.86	7.09±6.90	<0.001
Serum albumin (g/dL)	3.58±0.50	3.50±0.52	3.62±0.50	0.494
Hypoalbuminemia – n (%)	261 (48.0)	99 (46.3)	162 (49.0)	0.214
Serum ferritin (ug/dL)	1097.03±1300.67	829.41±634.96	1255.88±1547.51	0.006
CRP (mg/dL)	9.51±9.29	8.16±8.27	10.33±9.77	0.008
Acidemia – n (%)	43 (7.9)	5 (2.3)	38 (11.5)	0.001
Lactate level (mg/dL)	14.53±10.02	13.95±9.62	14.86±10.24	0.344
Nephrotoxins – n (%)	78 (14.3)	28 (13.1)	50 (14.7)	0.715
ICU admission – n (%)	120 (22.1)	33 (15.4)	87 (25.7)	0.009
Mechanical ventilation – n (%)	69 (12.7)	18 (8.4)	51 (15.2)	0.029
Vasopressor use – n (%)	18 (3.3)	4 (1.9)	14 (4.2)	0.160
ARDS – n (%)	56 (10.4%)	16 (7.5)	40 (12.1)	0.124
COVID-19 treatment				
Hydroxychloroquine – n (%)	156 (28.7)	61 (28.5)	95 (28.8)	0.728
Lopinavir/ritonavir – n (%)	199 (36.6)	76 (35.5)	123 (37.3)	0.893
Tocilizumab – n (%)	18 (3.3)	6 (2.8)	12 (3.6)	0.685
Corticosteroids – n (%)	140 (25.7)	43 (20.0)	97 (29.4)	0.035
Remdesivir – n (%)	47 (8.6)	12 (5.6)	35 (10.6)	0.064
AKI – n (%)	330 (60.6)			
Persistent AKI – n (%)			166 (50.3)	
KDIGO stage 1 – n (%)			109 (33.0)	
KDIGO stage 2 – n (%)			46 (13.9)	
KDIGO stage 3 – n (%)			184 (55.8)	
RRT requirement – n (%)	53 (9.7)		53 (16.1)	
LOS in hospital (days)	31.9±43.14	29.11±41.09	33.62±44±31	0.238
Discharge SCr (mg/dl)	0.99±0.67			
In-hospital mortality – n (%)	76 (14.0)	15 (7.0)	61 (18.5)	<0.001

AKI – acute kidney injury; ARDS – acute respiratory distress syndrome; CKD – chronic kidney disease, COPD – chronic obstructive pulmonary disease; COVID-19 – Coronavirus disease 2019; CRP – C reactive protein; CVD – cardiovascular disease. eGFR – estimated glomerular filtration ratio; ICU – intensive care unit; KIDGO – Kidney International Disease Global Outcome; LOS – length of hospital satay; NL ratio – neutrophil/lymphocyte ratio; RAAS – Renin-angiotensin-aldosterone system; RRT – renal replacement therapy; SCr – serum creatinine.

The majority of patients admitted were male (n=298, 54.8%), with a mean age of 68.9±17.9 years. Arterial hypertension was the most common comorbidity (n= 345, 63.4%), followed by cerebrovascular disease and diabetes (n= 175, 32.2% and n=146, 26.8%, respectively). Baseline creatinine was only estimated with MDRD in 29 patients (5.3%). Mean baseline SCr was 0.98±0.44 mg/dL, mean GFR was 75.68±24.89 mL/min/1.73m^2^ and 103 patients had CKD (18.9%). Two hundred and twenty-six patients were medicated with RAAS inhibitors. During hospital stay, 78 patients received nephrotoxins, such as NSAIDS, radiocontrast, vancomycin, or aminoglycosides.

At hospital admission, mean SCr was 1.32±1.47 mg/dL, mean hemoglobin level was 12.68±2.29 g/dL (41.2% patients had anemia), mean NL ratio was 6.36±6.28, mean serum albumin was 3.58±0.50 g/dL (48% patients had hypoalbuminemia), mean serum ferritin was 1097.03±1300.67 µg/L, mean CRP was 9.51±9.29 mg/dL, mean lactate level was 14.53±10.02 mg/dL, and 43% of patients were acidemic.

Concerning treatment, a vast majority of patients were medicated with lopinavir/ritonavir (n=199, 36.6%), hydroxychloroquine (n=156, 28.7%), and corticosteroids (n=140, 25.7%), whereas only 47 patients (8.6%) were medicated with remdesivir and 18 patients (3.3%) were treated with tocilizumab.

More than 20% of hospitalized patients (n=120) required ICU admission mostly due to respiratory failure, 56 patients fulfilled ARDS criteria, 69 patients required mechanical ventilation, and 18 patients required vasopressor support.

### Acute kidney injury

In this cohort of SARS-COV-2 infected patients, 60.6% developed AKI during hospital stay (n=330). Of these, 50.3% (n=166) presented pAKI defined as AKI persisting for more than 48 hours or requiring renal replacement therapy (RRT). According to AKI severity, most patients were KDIGO stage 3 (n=184, 55.8%), followed by KDIGO stage 1 (n=109, 33.0%), and KDIGO stage 2 (n=46, 13.9%); 16.1% of AKI patients required RRT.

Patients with AKI were older (71.73±17.03 vs 64.23±18.38 years, p<0.001), were more likely to have previous comorbidities - arterial hypertension (72.1 vs 50.0%, p<0.001), diabetes (31.2 vs 20%, p=0.016), cerebrovascular disease (39.1 vs 21.5, p<0.001), CKD (26.1 vs 7.9%, p<0.001) - and to be medicated with RAAS inhibitors (47.6 vs 32.2%, p=0.002). Mean baseline SCr was higher in AKI patients (1.03±0.44 vs 0.89±0.42, p<0.001). Patients with BCRSS score higher than 2 more frequently developed AKI (32.9 vs 10.3% p= 0.039). At hospital admission, patients with AKI had higher SCr (1.60±1.76 vs 0.87±0.55, p<0.001), higher NL ratio (7.09±6.90 vs 5.15±4.86, p<0.001), and were more likely to be acidemic (11.5 vs 2.3%, p=0.001). AKI patients required more ICU admission (25.7 vs 15.4%, p<0.009) and mechanical ventilation (15.2 vs 8.4%, p<0.029). There was no difference on vasopressor used, fulfilled ARDS criteria, and drug treatment for SARS-COV-2 infection between patients with and without AKI.

On a multivariate analysis (Table 2), CKD (adjusted OR 5.022; 95%CI 1.606-15.702, p=0.006) and serum ferritin (adjusted OR 1.001; 95%CI 1.000-1.001, p=0.009) were independent predictors of AKI.

**Table 2:** Univariate and multivariate analysis of factors predictive of AKI in COVID-19 patients

Characteristic	AKI			
	Unadjusted OR (95% CI)	P-value	Adjusted OR (95% CI)	P-value
Age	1.024 (1.014-1.034)	0.000	1.008 (0.987-1.030)	0.444
Gender (male)	1.182 (0.835-1.674)	0.345		
Co-morbidities				
Hypertension	2.158 (1.506-3.092)	0.000	1.508 (0.750-3.031)	0.249
Diabetes	1.644 (1.093-2.473)	0.017	1.624 (0.780-3.383)	0.195
CVD	2.123 (1.431-3.151)	0.000	1.096 (0.513-2.242)	0.812
CKD	3.804 (2.187-6.617)	0.000	5.022 (1.606-15.702)	0.006
COPD	1.000 (0.0594-1.684)	1.000		
Cirrhosis	1.053 (0.613-1.811)	0.851		
Neoplasia	0.932 (0.582-1.492)	0.769		
Brescia Score	1.434 (1.143-1.798)	0.002	1.088 (0.806-1.467)	0.582
Hemoglobin	0.955 (0.885-1.031)	0.241	1.065 (0.980-1.033)	0.139
Anemia	1.145 (0.803-1.631)	0.455		
NL ratio	1.066 (1.027-1.107)	0.001	1.065 (0.980-1.033)	0.139
Serum albumin	1.600 (0.431-5.943)	0.483		
Serum ferritin	1.000 (1.000-1.001)	0.010	1.001 (1.000-1.001)	0.009
LDH	1.001 (1.000-1.003)	0.057		
CRP	1.027 (1.007-1.048)	0.009	0.995 (1.000-1.001)	0.804
Acidemia	4.702 (1.816-12.178)	0.001	11.095 (1.377-89.413)	0.024
Lactate level	1.010 (0.989-1.031)	0.349		

CKD – chronic kidney disease, COPD – chronic obstructive pulmonary disease; CRP – C reactive protein; CVD – cardiovascular disease; LDH – lactate dehydrogenase; NL ratio – neutrophil/lymphocyte ratio.

### Persistent AKI vs transient AKI

Regarding AKI duration, patients with pAKI were older (71.45±17.15 vs 67.73±18.31 years of age, p=0.058) had more arterial hypertension (71.7 vas 59.1%, p=0.017) and more nephrotoxin exposure (19.9 vs 10.4%, p=0.015). Diabetes (30.7 vs 23.8%, p=0.157), cerebrovascular disease (39.8 vas 26.8%, p=0.013), CKD (24.7 vs 17.7%, p=0.112), and medication with RAAS inhibitors (48.2 vs 41.5%, p=0.165) were more common in patients with pAKI than in patients with transient AKI.

Patients with pAKI had higher SCr level (1.71±2.37 vs 1.25±0.68, p=0.026) and more acidemia (11.4 vs 6.1%, p=0.085) at hospital admission. No differences were observed on severity of AKI between groups: KDIGO 1- 28.9 vs 34.1%, KDIGO 2- 18.1 vs 8.5%, KDIGO 3- 53.0 vs 56.7%).

These data are shown in Table 3.

**Table 3:** Characteristics of patients with persistent and transient AKI

Characteristic	Transient AKI (n=164)	Persistent AKI (n=166)	p-value
Age (year)	67.73±18.31	71.45±17.15	0.058
Gender (male) – n (%)	90 (54.9)	97 (58.4)	0.515
Co-morbidities – n (%)			
Hypertension	97 (59.1)	119 (71.7)	0.017
Diabetes	39 (23.8)	51 (30.7)	0.157
CVD	44 (26.8)	66 (39.8)	0.013
CKD	29 (17.7)	41 (24.7)	0.112
COPD	20 (12.2)	24 (14.5)	0.545
Cirrhosis	7 (4.3)	8 (4.8)	0.586
Neoplasia	23 (14.0)	23 (13.9)	0.965
RAAS inhibitors – n (%)	68 (41.5)	80 (48.2)	0.165
Baseline SCr (mg/dL)	0.97±0.34	1.03±0.45	0.151
Baseline eGFR (mL/min/1.73m2)	75.58±24.04	72.15±25.55	0.211
Brescia Score ≥ 2	19 (11.59)	33 (19.9)	0.005
Laboratory			
Admission SCr (mg/dL)	1.25±0.68	1.71±2.37	0.026
Hemoglobin (g/dL)	12.71±2.45	12.58±2.30	0.621
Anemia – n (%)	64 (39.0)	74 (44.6)	0.306
NL ratio	6.57±6.79	6.96±6.57	0.599
Serum albumin (g/dL)	3.57±0.51	3.62±0.50	0.825
Hypoalbuminemia – n (%)	69 (42.1)	82 (49.4)	0.850
Serum ferritin (ug/dL)	1231.38±1608.02	1097.97±1176.80	0.523
CRP (mg/dL)	9.65±10.02	9.59±8.49	0.925
LDH (mg/dL)	335.34±149.55	351.26±172.83	0.373
Acidemia – n (%)	10 (6.1)	19 (11.4)	0.085
Lactate level (mg/dL)	15.07±8.94	13.56±6.60	0.103
Nephrotoxins – n (%)	17 (10.4)	33 (19.9)	0.015
ICU admission – n (%)	32 (19.5)	58 (34.9)	0.002
Mechanical ventilation – n (%)	19 (11.6)	33 (19.9)	0.093
Vasopressor use – n (%)	3 (1.8)	12 (7.2)	0.028
ARDS – n (%)	13 (7.9)	30 (18.1)	0.014
COVID-19 treatment			
Hydroxychloroquine – n (%)	39 (23.8)	58 (34.9)	0.021
Lopinavir/ritonavir – n (%)	56 (34.1)	71 (42.8)	0.107
Tocilizumab – n (%)	3 (1.8)	7 (4.2)	0.195
Corticosteroids – n (%)	41 (25.0)	52 (31.3)	0.135
Remdesivir – n (%)	16 (9.8)	16 (9.6)	0.985
KDIGO stage			
KDIGO stage 1 – n (%)			
56 (34.1)			
48 (28.9)	0.038		
KDIGO stage 2 – n (%)	14 (8.5)	30 (18.1)	
KDIGO stage 3 – n (%)	93 (56.7)	88 (53.0)	
LOS in hospital (days)	29.29±38.00	37.74±46.89	0.073
LOS in ICU (days)	3.79±9.27	7.32±13.73	0.022
Discharge SCr (mg/dL)	0.97±0.69	1.17±0.88	0.026
In-hospital mortality – n (%)	20 (12.2)	35 (21.1)	0.030

AKI – acute kidney injury; ARDS – acute respiratory distress syndrome; CKD – chronic kidney disease, COPD – chronic obstructive pulmonary disease; COVID-19 – Coronavirus disease 2019; CRP – C reactive protein; CVD – cardiovascular disease; eGFR – estimated glomerular filtration ratio; ICU – intensive care unit; KIDGO – Kidney International Disease Global Outcome; LDH – lactate dehydrogenase; LOS – length of hospital stay; NL ratio – neutrophil/lymphocyte ratio; RAAS – Renin-angiotensin-aldosterone system; RRT – renal replacement therapy; SCr – serum creatinine.

### Outcomes

Mean length of hospital stay was 31.9±43.14 days and no statistical difference was found between AKI and non-AKI groups (33.62±44±31 vs 29.11±41.09, p=0.238). The mean SCr level at hospital discharge was 0.99±0.67mg/dL.

Overall, in-hospital mortality was 14.0% (n=76), and mortality was higher in AKI patients (18.5 vs 7.0%, p<0.001). On a multivariate analysis (Table 4), AKI was not an independent predictor of mortality (adjusted OR 0.88; 95%CI 0.32-2.44, p=0.808) but a subgroup analysis revealed that pAKI was an independent predictor of mortality (adjusted OR 10.57; 95%CI 2.45-45.49, p=0.002). Age (adjusted OR 1.072; 95%CI 1.011-1.137, p=0.002) and lactate level (adjusted OR 1.077; 95%CI 1.011-1148, p=0.002) were also independent predictors of mortality.

**Table 4:** Univariate and multivariate analysis of factors predictive of mortality in COVID-19 patients

Characteristic	Mortality			
	Unadjusted OR (95% CI)	P-value	Adjusted OR (95% CI)	P-value
Age	1.057 (1.037-1.078)	0.000	1.072 (1.011-1.137)	0.002
Gender (male)	0.904 (0.556-1.470)	0.685		
Co-morbidities				
Hypertension	2.032 (1.160-3.560)	0.013	4.257 (0.637-28.443)	0.135
Diabetes	1.048 (0.609-1.803)	0.866		
CVD	2.741 (1.675-486)	0.000	0.557 (0.171-1.811)	0.330
CKD	3.509 (2.065-5.965)	0.000	1.078 (0.328-3.541)	0.901
COPD	0.913 (0.433-1.927)	0.812		
Cirrhosis	0.950 (0.432-2.092)	0.899		
Neoplasia	2.526 (1.442-4,425)	0.001	1.069 (0.202-5.666)	0.938
Brescia score	1.334 (1.088-1.636)	0.006	1.052 (0.599-1.848)	0.859
Hemoglobin	0.787 (0.709-0.874)	0.000	0.829 (0.637-1.079)	0.162
Anemia			0.972 (0.899-1.051)	0.447
NL ratio	1.050 (1.018-1.083)	0.002	0.977 (9.03-1.056)	0.570
Serum albumin	1.05 (1.000-1.001)	0.998		
Serum ferritin	1.000 (1.000-1.000)	0.009	1.001 (1.000-1.001)	0.005
LDH	1.002 (1.001-1.003)	0.000	1.002 (0.999-1.005)	0.258
CRP	1.015 (0.990-1.041)	0.239		
Acidemia	1.861 (0.874-3.964)	0.107		
Lactate level	1.064 (1.034-1.094)	0.000	1.077 (1.011-1148)	0.002
ICU admission	0.931 (0.514-1.684)	0.813		
AKI	2.779 (1.534-5.035)	0.001	0.88 (0.32-2.44)	0.808
KDIGO Stage	1.03 (0.75-1.40)	0.863		
AKI 48h or RRT	5.699 (3.286-9.883)	0.000	10.57 (2.45-45.49)	0.002

AKI – acute kidney injury; CKD – chronic kidney disease, COPD – chronic obstructive pulmonary disease; CRP – C reactive protein; CVD – cardiovascular disease; eGFR – estimated glomerular filtration ratio; ICU – intensive care unit; KIDGO – Kidney International Disease Global Outcome; LDH - lactate dehydrogenase; NL ratio – neutrophil/lymphocyte ratio; RRT – renal replacement therapy.

## Discussion

In this retrospective analysis we report a high incidence of AKI associated with COVID-19, and that persistent AKI was independently associated with mortality.

AKI development in COVID-19 patients has been reported in previous studies. The incidence of AKI reported in hospitalized patients with COVID-19 ranges from 5.1 to 75.0%^6^^,^ 16 ^-^ 24. The wide range of AKI incidence may be explained by differences on demographics, comorbidities, and disease severity because almost all studies used the KDIGO definition.

The studies that reported a lower incidence of AKI on COVID-19 patients, as in the study by Wang et al., who reported an incidence of 5.1%^17^ and Cui et al., who reported an incidence of AKI of 18.1%^19^, had younger patients and with fewer comorbidities than our patients. Fisher et al., in a retrospective study of 4609 patients, reported a incidence of AKI in COVID 19 patients similar to our study: 56.9%^25^. Fominskiy et al., in a retrospective observational study of 99 patients with COVID-19, reported a 75% of AKI incidence^20^, but they only analyzed patients requiring mechanical ventilation.

The largest cohort of hospitalized patients with COVID-19, which included 5449 patients, reported an AKI incidence of 36.6% which developed mainly early in the course of COVID-19 infection and 46.5% of AKI patients had AKI KDIGO stage 1^6^. This is in accordance with our study, in which most patients developed AKI within the first 48 hours. Most studies have reported that most patients present with lower (KDIGO 1) or higher severity (KDIGO 3)^20^^-^ 22. In our study, most patients were KDIGO stage 3. There was no difference in AKI severity between transient and persistent AKI. In fact, the prevalence of stage 3 AKI was slightly higher in tAKI, which could be due to dehydration secondary to vomiting or diarrhea, which could resolve rapidly after hospital admission. Some patients probably had AKI before hospital admission, as reflected by a higher SCr at hospital admission compared with baseline SCr.

The etiology of AKI in COVID-19 patients appears to be multifactorial. It can be related to fluid balance disturbances secondary to gastrointestinal symptoms (nausea, vomiting and diarrhea), renal venous congestion secondary to myocardiopathy or acute viral myocarditis^7^, toxic tubular damage following cytokine release syndrome or rhabdomyolysis^8^, direct cytopathic effect of SARS-CoV-2^26^, endothelitis, thrombotic events and intravascular coagulation^27^^,^ 28, nephrotoxicity from drugs such as lopinavir/ritonavir, nucleoside analogues, remdesivir, tenofovir, chloroquine phosphate and hydroxychloroquine sulfate^29^, and interaction between SARS-COV-2 and angiotensin II receptors (apparently the patients with low D allele polymorphism have high mortality)^7^.

In our study, CKD and serum ferritin were independent predictors of AKI development. In previous studies, age, CKD, hypoalbuminemia, lymphopenia and neutrophil/lymphocytes ratio, lactate dehydrogenase, d-dimers, C-reactive protein, and need for mechanical ventilation or vasopressor support were reported as independent predictors of AKI devolopment^6^^,^ 16 ^,^ 20 ^,^ 25 ^,^ 30. Despite the considerable focus on the use of RAAS inhibitors and severity of COVID-19 and a recent study of Soleimani et al. which reported the association of RAAS inhibitors and AKI development, this was not found in our cohort^31^. Interestingly, in that study, the discontinuation of RAAS was associated with a greater risk of mortality, of invasive ventilation, and of AKI. Another two studies found that RAAS inhibitors were not associated with increased mortality in COVID-19 patients^32^^-^ 33 and, in contrast, one of those studies found that the discontinuation of RAAS inhibitors was associated to a high mortality of COVID-19 patients^26^.

Other studies have tried to find biomarkers predictive of AKI in COVID-19 patients. Azam et al., in a study with 352 hospitalized COVID-19 patients, of which 91 had AKI, reported that soluble urokinase receptor (suPAR) predicted AKI development^34^. Husan-Syed et al. analyzed the utility de urinary biomarkers to predict AKI in COVID-19 patients. They found that alfa-1-microglobulin excretion was higher in patients who developed AKI and that AKI patients with increased [TIMP-2]•[IGFBP7] levels seemed to have worse prognosis^35^.

Previous studies demonstrated a higher rate of mortality in COVID-19 patients with AKI. Cheng et al., in a prospective cohort of 701 hospitalized patients, reported a higher risk of mortality in patients with more severe AKI, despite AKI incidence being only 5.1%^16^. Lim et al. studied 164 patients with COVID-19 and demonstrated that AKI KDIGO stage 3 was associated with higher mortality^30^. Cui et al., in a multicenter retrospective observational study of 116 COVID-19 patients, reported a greater mortality in patients with AKI (57.1 vs 12.6%, p=0.000)^19^. A recent study by Chan et al., in a larger cohort of 3993 hospitalized patients with COVID-19, found that AKI was associated to a higher mortality, as 50% of AKI patients died versus 8% of non-AKI (p<0.001)^22^.

Hirsch et al. described an important relationship between AKI and respiratory failure. First, they found that most of the cases of severe AKI occurred in close temporal proximity to intubation and mechanical ventilation and secondly, patients on ventilators had a higher AKI rate and more severe AKI stages^6^. Interestingly, in our cohort of patients the development of acidemia was a predictive factor for AKI but lactate level was not, which possibly reflected disease severity associated to COVID-19, mainly respiratory acidemia. In fact, the mean lactate level of our cohort was below 20 mg/dL. Some studies that only analyzed the mortality associated with AKI in critically ill COVID-19 patients reported higher mortality in those patients. Fominskiy et al., in a study with patients with COVID-19 admitted in the UCI requiring invasive mechanical ventilation, found that patients with AKI had 40% mortality and patients that required continuous RRT had a 50% mortality^20^. Xu et al., in a retrospective multicenter observational study with 671 patients with COVID-19 admitted in the UCI, reported a higher mortality at 28 days in patients with AKI (72 vs 42%, p<0.001)^21^.

In our study, AKI was not predictive of mortality in COVID-19 patients but the persistence of AKI for more than 48 hours was. None of the previously mentioned studies evaluated persistent AKI in mortality. According to previous studies in patients without COVID-19, persistent AKI affected mortality^36^^-^ 38. To date, we are not aware of any study that evaluated the real impact of AKI duration on vital prognosis of COVID-19 patients. Thus, the question of whether mortality associated with AKI in COVID-19 patients is mainly influenced by the duration of AKI or by AKI development itself remains to be clarified. In fact, this question is extremely important, as Rubin et al. analyzed 77 critically ill patients with COVID-19 and demonstrated that persistent AKI was present in 93% of these patients^39^. Another important point is the follow-up of COVID-19 patients with persistent AKI. These patients should have a reassessment of renal function and cardiovascular risk within 30 days of follow-up, as proposed by Kellum in patients with pAKI not associated with COVID-19^40^.

Previous studies in COVID-19 patients with AKI demonstrated that age, AKI severity, and high SOFA score were independent predictors of mortality^16^^,^ 18 ^,^ 19 ^,^ 22 ^,^ 25. In our study, Brescia score ³2 was not predictive of mortality, maybe because this score does not include age and lactate level, which were factors predictive of mortality in our cohort.

The current study has some noteworthy limitations. First, the single-center retrospective nature limits the generalizability of our results. We did not analyze laboratorial parameters that were available for majority of the patients, such as urinalysis, which might have added important diagnostic and prognostic information. Finally, we did not analyze the exact mechanisms contributing to AKI and mortality.

Nevertheless, our study has some important merits. This is one of the first studies to evaluate the impact of AKI duration on mortality in COVID-19 patients. AKI was defined and stratified according to the KDIGO classification using SCr criteria. Both AKI severity and AKI duration were assessed to evaluate their impact on prognosis. Despite the retrospective design, the variables studied were routinely recorded in daily practice.

## Conclusion

To conclude, we demonstrated that AKI was frequent in hospitalized patients with COVID-19 and that persistent AKI was independently associated with in-hospital mortality. Older age and higher lactate levels were also predictors of mortality in this cohort. This study highlights the need to improve early detection of AKI in order to initiate timely therapeutic strategies, as rapid recovery of renal function within 48 hours is associated with a better prognosis. The impact of AKI duration on the long-term follow up of COVID-19 patients remains to be determined.
